# Combined antihypertensive effect of unripe *Rubus coreanus* Miq. and *Dendropanax morbiferus* H. Lév. Extracts in 1 kidney-1 clip hypertensive rats and spontaneously hypertensive rats

**DOI:** 10.1186/s12906-021-03438-4

**Published:** 2021-10-28

**Authors:** Soyi Park, Ki Hoon Lee, Hakjoon Choi, Goeun Jang, Wan Seok Kang, Eun Kim, Jin Seok Kim, Chang-su Na, Sunoh Kim

**Affiliations:** 1 Central R&D Center, Bioresources and Technology (B&Tech) Co., Ltd., 257, Jebong-ro, Buk-gu, Gwangju, 61239 South Korea; 2 College of Korean Medicine, Dongshin University, 185 Geonjae-ro, Naju-si, Jeollanam-do 58245 Republic of Korea

**Keywords:** Enzymatically hydrolyzed *Dendropanax morbiferus* H. Lév., Unripe *Rubus coreanus* Miq., 1 kidney-1 clip hypertensive rat, Spontaneously hypertensive rat, Hypertension

## Abstract

**Background:**

We previously showed that enzymatically hydrolyzed *Dendropanax morbiferus* H. Lév. leaf (Hy-*DP*) and unripe *Rubus coreanus* Miq. (5-*u*RCK) extracts exhibit potent vasodilator effects on isolated aortic rings from rats partly through endothelium-dependent and endothelium-independent mechanisms. These two extracts have different mechanisms of action; however, their combined effect on antihypertensive activity has not been explored.

**Methods:**

The present study aims to investigate the effect of a chronic optimized mixture (HDR-2, composed of Hy-*DP* and 5-*u*RCK in a 2:1 mass ratio) on vascular tension and blood pressure in two different hypertensive rat models.

**Results:**

The results showed that HDR-2 concentration-dependently relaxed endothelium-intact and endothelium-denuded aortic rings precontracted with phenylephrine. Antihypertensive effects were assessed in vivo on a 1 kidney-1 clip (1 K-1C) rat model of hypertension and spontaneously hypertensive rats (SHRs). Acute HDR-2 treatment significantly decreased systolic blood pressure (SBP) 3 h posttreatment in both models. Chronic HDR-2 administration also significantly decreased SBP in the hypertensive rat models. Moreover, HDR-2 increased eNOS protein expression and phosphorylation levels in the aorta.

**Conclusion:**

Chronic HDR-2 administration may effectively improve vascular function by decreasing plasma angiotensin-converting enzyme (ACE) activity and AngII levels. HDR-2 significantly improved acetylcholine (ACh)-induced aortic endothelium-dependent relaxation and affected sodium nitroprusside (SNP)-induced endothelium-independent relaxation in SHRs.

## Background

High blood pressure is one of the major risk factors for the development of vascular diseases such as atherosclerosis, coronary heart disease and stroke [1], and several studies have reported cardiovascular risks associated with elevated blood pressure [2, 3]. Hypertension, a global public health risk, is highly associated with heart disease, stroke, kidney failure, premature death and disability [4]. Currently, many antihypertensive therapeutic drugs have been developed and are now available for use in clinical treatment, including thiazide diuretics, β-blockers, renin-angiotensin-aldosterone system (RAAS) inhibitors and Ca^2+^ channel blockers. However, side effects have been reported with these treatments. Thiazide diuretics could potentially result in insulin resistance, dyslipidemia and hyperuricemia, which would accelerate diabetes progression in patients with obesity and metabolic syndromes [5]. Traditional β-blockers also appear to have a significantly higher risk of diabetes. RAAS inhibitors, by angiotensin-converting enzyme (ACE) inhibitors or an angiotensin receptor blocker (ARB) along with Ca^2+^ channel blockers, appear to have improved safety properties, but some of them still have various side effects, such as coughing, taste disturbance and skin rashes [6]. Clearly, future investigations for novel and safe antihypertensive drugs are still required.

*Dendropanax morbiferus* H. Lév. is an endemic tree species in Korea belonging to the family Araliaceae, and extracts are traditionally used to treat various diseases [7]. Active chemicals in *D. morbiferus* leaf extract (*DP*) have also various biological effects [8–11]. Furthermore, we reported the change in the content of quercetin, a marker chemical, depending on the parts, harvest season, and extraction methods of *D. morbiferus* for quality control [12]. Quercetin and its metabolites, such as 3-(3-hydroxyphenyl)propionic acid (3HPPA), 3,4-dihydroxyphenylacetic acid (DHPA) and 4-methylcatechol (4MC), administered orally are well known to be able to decrease arterial blood pressure [13–15]. Another our previous study reported that we developed a technique to increase _L_-arginine and γ-aminobutyric acid (GABA) levels of *DP* by enzymatic hydrolysis (producing Hy-*DP*) [16]. _L_-Arginine- and GABA-rich extracts have been demonstrated to reduce blood pressure in clinical studies [17, 18]. GABA, contained in various foods, has been suggested to be a major component regulating cardiovascular function and hypertension [19–23]. Furthermore, in our previous study, *DP* and Hy-*DP* dose-dependently relaxed endothelium-intact aortic rings precontracted with phenylephrine (PHE). Between them, Hy-*DP* exhibited the more potent vascular relaxant effect [24].

The *Rubus* genus comprises thousands of species of blackberries and raspberries grown worldwide, and species in this genus have been investigated as novel therapeutic agents for metabolic syndrome through their beneficial effects [25]. One such species, *Rubus coreanus* Miquel, which is native to eastern Asia, has been used as a traditional alternative medicine to manage impotence, spermatorrhea, enuresis, asthma and allergic diseases [26]. The major active chemical in *R. coreanus* is ellagic acid, which has multi-biological properties [27, 28]. According to a report, it is suggested that the concentration of ellagic acid, the main active compound of *R. coreanus*, is higher in immature stage than in the mature stage [29]. In particular, comparing the efficacy of each extracts according to various extraction methods of unripe *R. coreanus*, 5% ethanol extract of unripe *R. coreanus* (5-*u*RCK) has the best effect on hyperlipidemia [30, 31], obesity [32, 33] and hypertension [24] reported, and interestingly, it was confirmed that the content of ellagic acid was the highest through this extraction method [24, 31, 33]. Thus, our previous studies in animal models demonstrated that 5-*u*RCK exerts antihypercholesterolemic, antiobesity and vasorelaxant effects.

Very recently, we scientifically validated that Hy-*DP* and 5-*u*RCK are involved in endothelial-dependent and endothelial-independent relaxation in the aortic ring [24]. Furthermore, we screened for various efficacies (ACE activity, cGMP dependent-PDE activity, cAMP dependent-PDE activity, and vasorelaxation) in mixtures of various mixing ratios (1:0, 1:1, 1:2, 1:3, 2:1, 3:1 and 0:1) of Hy-*DP* and 5-*u*RCK. Interestingly, when Hy-*DP* and 5-*u*RCK were mixed in a specific ratio of 2:1 (producing HDR-2), the greatest vasodilatory effect was shown. However, more research is required to evaluate the detailed mechanisms in experimental animal models of hypertension. In this work, therefore, we intend to conduct further studies to elucidate the detailed mechanism using two different hypertensive animal models, namely, the spontaneously hypertensive rat (SHR) model, an established model of genetic hypertension, and the 1 kidney-1 clip (1 K-1C) Goldblatt rat model of hypertension.

## Methods

### Reagents

All the chemicals were purchased from Sigma-Aldrich (St. Louis, MO, USA) or Tocris (Ellisville, MO, USA). All the media components were purchased from Invitrogen, Inc. (Grand Island, NY, USA).

### Preparation of extracts

The unripe *R. coreanus* fruits used in this study were collected (May 2017) in Gochang-gun (Jeollabukdo, Korea). Extracts of the unripe *R. coreanus* were described in our previous study [24, 30–33]. The dried leaves of *D. morbiferus* used in this study were collected (December 2017) in Gangjin-gun (Jeollanamdo, Korea) and Hy-*DP* extraction was performed as described in our previous study [16, 24]. HDR-2 consisted of an optimized mixture of compounds derived from Hy-*DP* and 5-*u*RCK.

### Animals

Healthy male Sprague–Dawley rats (S-D rats, *n* = 20) were used for the ex vivo aortic ring experiments, male Wistar rats (*n* = 42, weighing 250 to 300 g each) were used for the 1 K-1C hypertensive rat model experiments, and five-week-old male SHRs (*n* = 20) and normotensive male Wistar Kyoto rats (WKY, *n* = 5) were used for the blood pressure experiments. All animals were purchased from Central Lab Animal, Inc. (Seoul, Republic of Korea). The experiment was conducted according to the international guidelines [34] and was approved by the institutional animal care and use committee (IACUC) of the B&Tech Co., Ltd., Korea (Approval number: BT-002-2018).

### Induction of 1-kidney, 1-clip Goldblatt hypertensive (1 K-1C) rats

Induction of renal hypertension was carried out in rats according to the 1 K-1C rat model originally described by Goldblatt et al. [35] and later modified by Laffan et al. [36] and McCaa et al. [37]. Briefly, male Wistar rats (3 months old, weighing 200–250 g) were used for induction of the 1 K-1C model. The rats were anesthetized with intramuscular injection of ketamine (40 mg/kg), xylazine (8 mg/kg) and chlorpromazine (4 mg/kg). The renal artery was exposed through a laparotomy and dissected from the renal vein. A U-shaped silver clip with an internal diameter of 0.20 mm was placed around the left renal artery as close as possible to the aorta. The clip was then turned such that the slit opening faced the abdomen, and the contralateral kidney was removed without the adrenal gland (Goldblatt rats). Sham operation for the control for the 1 K-1C model was performed in a similar manner, including the removal of one kidney but not the clipping of the remaining renal artery (1 K-NC). Surgical mortality was below 5%, and the 5-week mortality rate was 15%. Three weeks after the surgical operation, 1 K-1C rats were used for blood pressure monitoring after confirming the presence of high blood pressure (systolic blood pressure > 160 mmHg) using a tail-cuff method. Then, the rats were randomly divided into five groups: a control group (2 K-NC, *n* = 7); a sham-operated group (1 K-NC, *n* = 7); a 1 K-1C group (*n* = 7), a 1 K-1C + HDR-2 (150 mg/kg/day by oral gavage) group (*n* = 7), a 1 K-1C + HDR-2 (300 mg/kg/day by oral gavage) group (*n* = 7), and a 1 K-1C + mixture of _L_-arginine and GABA (4:6 ratio; 10 mg/kg/day by oral gavage) group (*n* = 7). HDR-2 was dissolved in saline, and the control group received only saline. Then, the dosing volume was calculated from the body weight of rats (0.5 mL per rat).

### Experimental groups

Five WKY and twenty SHR were randomly assigned to five groups (*n* = 5): a control WKY group and a control SHR group, both of which received water as vehicle, and SHR groups treated with HDR-2 (150 or 300 mg/kg/day by oral gavage) or a mixture of _L_-arginine and GABA (4:6 ratio; 10 mg/kg/day by oral gavage) for 5 weeks. HDR-2 was dissolved in saline, and the control group received only saline. Then, the dosing volume was calculated from the body weight of rats (0.5 mL per rat). HDR-2 treatment was stopped 24 h before the end of the experiment to study the long-term effects of HDR-2 without involving the effects of acute administration. Food and water intake were recorded daily for all groups. During the experimental periods, rats had free access to tap water and chow. The body weight was measured every week.

### Tissue collection and cardiac and renal weight indices

When the experimental period was complete, animals were fasted for 18 h and anesthetized with 2.5 mL/kg pentobarbital (intraperitoneal injection; i.p.), followed by decapitation (with rats held horizontally during blood collection to minimize contamination of the blood sample by gastric juice). The kidneys and ventricles were then removed and weighed. The heart was divided into the right ventricle and left ventricle plus septum. The heart weight index, kidney weight index, liver weight index and spleen weight index were calculated by dividing the heart weight, kidney weight, liver weight and spleen weight by the body weight (BW). The thoracic aorta and lungs were excised for further analyses and stored at − 80 °C until analysis. The thoracic aortae were isolated immediately and used for ex vivo vascular function studies.

### Measurement of systolic blood pressure and heart rate in unanesthetized rats

The average systolic blood pressure of the animals was measured by the tail-cuff method using a noninvasive blood pressure system (NIBP system, Panlab/Harvard Apparatus, Barcelona, Spain) before all treatments and every subsequent week after the treatment started. Briefly, all animals were acclimated to the restraint condition before measurement. They were immobilized in a prewarmed chamber (28–30 °C) for at least 30 min before each blood pressure measurement was carried out. At least six to seven successive measurements were recorded, and the average values of these readings are reported. All SBP measurements were carried out at the same time of day.

### Ex vivo experiments for measurement of vascular responsiveness

After anesthesia with isoflurane, S-D rat or SHR thoracic aortae were resected and placed in Krebs’ buffer solution bubbled with 5% CO_2_ and 95% O_2_. The contractile activity was measured according to the same method as in our former study [24]. Upon attaining a constriction plateau, vasodilation ability was verified by treatment with acetylcholine chloride (ACh, 10^− 9^–10^− 3^ M) or sodium nitroprusside (SNP, 10^− 9^–10^− 3^ M) in the endothelium-denuded rings.

### Cell preparation

Cultured hippocampal neurons were proceeded in the same manner as described in previous study [24]. Murine RAW 264.7 macrophages (40071) were obtained from the Korea Cell Line Bank (KCLB, Seoul, Korea) and human umbilical vein endothelial cells (HUVECs) were obtained from the American Type Culture Collection (ATCC, Manassas, VA, USA). iNOS and eNOS were also measured in the same method as in our former study [24].

### Gene expression analysis

Gene expression in cultured cells was analyzed by RT-PCR, as previously described [24]. The specific primers used in this study (eNOS, nNOS, and iNOS) were also analyzed using the same primers used in our previous study [24].

### NO production assay

The total NO (nitrite; NO_2_^−^ and nitrate; NO_3_^−^) levels in cell culture supernatants and serum were determined using the Griess reaction. Cell culture supernatants were collected 3 h after treatment with different compounds. Blood samples were collected at the end of the experiment from all rats by cardiac puncture. The plasma samples (100 μL) were mixed with zinc sulfate (50 μL, final concentration 15 mg/mL) and then centrifuged at 1000×g for 10 min. The supernatant (100 μL) was mixed with vanadium (III) chloride (100 μL, final concentration 8 mg/mL) and added to 2% sulfanilamide in 5% H_3_PO_4_ solution (50 μL) and 0.1% *N*-(1-naphthyl)ethylenediamine (50 μL). After incubation at 37 °C for 30 min, the absorbance was measured at 540 nm. The measurements were performed in triplicate, and the NO concentration (μM) was expressed as the means ± standard deviation (SD).

### Determination of plasma and lung ACE activity

ACE activity in serum and lung tissue was measured by using N-Hippuryl-His-Leu (HHL) as a substrate. The serum samples (50 μL) were mixed with 0.1 M sodium borate buffer (pH 8.3, 100 μL) containing 300 mM NaCl and 5 mM HHL (50 μL). The mixture was incubated at 37 °C for 45 min, and then the enzymatic reaction was stopped with the addition of 2 N HCl solution (200 μL). The reaction mixture was separated with ethyl acetate (2 mL). The upper ethyl acetate layer (1.5 mL) was concentrated and dissolved with 1 M NaCl solution (1 mL). The absorbance was measured at 228 nm, and the hippuric acid content in the serum and lung was calculated using a calibration curve of the hippuric acid standard.

### Determination of serum angiotensin II content

Plasma concentrations of angiotensin II (AngII) were quantified by an ELISA kit (AngII ELISA, Cayman, Ann Arbor, MI, USA) based on the manufacturer’s instructions. The plasma sample was added to a plate coated with AngII antibody, and then, biotinylated AngII solution was directly added to the plate, which was incubated for 2 h. The plate was washed with wash buffer, the buffer was removed, and the resulting contents were incubated with streptavidin peroxidase conjugate for 50 min at room temperature. After adding chromogen substrate, the plate was incubated for 10 min, and the absorbance was read at 450 nm.

### Protein extraction and immunoblot assays

Samples of aorta tissue were washed three times with cold PBS before being lysed in RIPA lysis buffer (10 mmol/L Tris-HCl, pH 7.5; 1% NP-40; 0.1% sodium deoxycholate; 0.1% SDS; 150 mmol/L NaCl; and 1 mmol/L EDTA) supplemented with 1× protease and phosphatase inhibitor cocktail (Thermo, Fremont, CA, USA) on ice. The separated proteins were transferred onto a nitrocellulose membrane. The anti-eNOS (1:100), anti-phospho-eNOS (1:100, Ser^1177^) and anti-β-actin (1:3000) antibodies and secondary antibodies (1:10000) were obtained from Cell Signaling Technology (Beverly, MA, USA). Immunoreactive protein bands were visualized using a ChemiDoc XRS+ System (Bio-Rad) and quantified with Gel Pro Analyzer software (Silk Scientific, Inc., Orem, UT, USA). The internal control, β-actin, was used to normalize differences due to loading variations.

### Statistical analysis

Results are presented as the mean and standard error of the mean (SEM) or standard deviation (SD). Data were analyzed by Student’s t-test or two-way analysis of variance (ANOVA) with GraphPad Prism version 8.0.0 for Windows (GraphPad, Inc., San Diego, California, USA) software programs. Differences at the *p* < 0.05 level were considered statistically significant.

## Results

### Effects of HDR-2 on isolated rat aortic rings precontracted with PHE

**Fig. 1:**
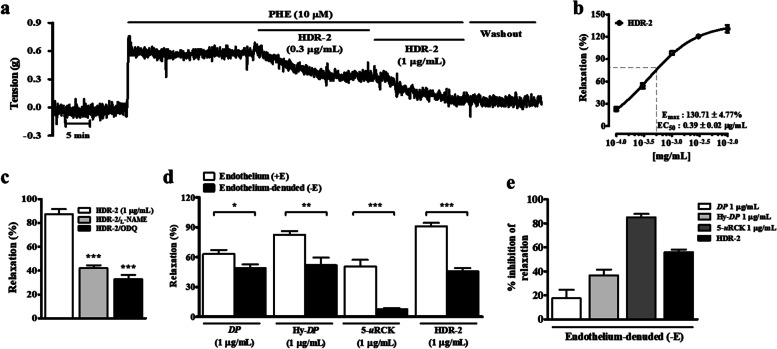
Effects of HDR-2 on thoracic aorta function. Representative traces of vascular relaxant responses induced by HDR-2 (**a**) in rat thoracic aortae precontracted with 10 μM PHE. Percent relaxation in response to increasing concentrations of HDR-2 (**b**) in aortic rings from SD rats. Lack of effect of _L_-NAME and ODQ on rat thoracic aorta relaxation induced by HDR-2 (**c**). Vasorelaxant effects of *DP*, Hy-*DP*, 5-*u*RCK and HDR-2 in thoracic aortae with denuded endothelia (−E) and intact endothelia (+E) (**d**). Inhibition of the vasorelaxant effects of *DP*, Hy-*DP*, 5-*u*RCK and HDR-2 in endothelium-free thoracic aortae precontracted with 10 μM PHE (e). The relaxation (%) values (mean ± SEM) are relative to the basal (submaximal) relaxation levels measured before PHE treatment, which were taken to be 100%. ^*^P < 0.05, ^**^P < 0.01 and ^***^P < 0.001 vs the CTL group

In our previous study, 5-*u*RCK exhibited an endothelium-dependent vasodilator effect, while *DP* induced a partial endothelium-dependent vasodilator effect. Additionally, we showed that Hy-*DP* has a stronger effect than *DP* and that Hy-*DP* improves impaired endothelium-dependent vasorelaxation in the aorta in SD rats [24]. In particular, the most significant effect was observed when Hy-*DP* and 5-*u*RCK were mixed at a 2:1 ratio (HDR-2). As shown in Fig. 1a and b, HDR-2 significantly relaxed endothelium-intact aortic rings precontracted with PHE (10 μM). As shown in Fig. 1c, HDR-2-induced relaxation was significantly inhibited by pretreatment with N^ω^-nitro-_L_-arginine methyl ester (_L_- NAME, 10 μM) and 1H-[1,2,4]oxadiazolo[4,3-a]quinoxalin-1-one (ODQ, 10 μΜ) in rat aortic rings with intact endothelia. However, the use of _L_-NAME and ODQ did not completely inhibit HDR-2-induced relaxation. A comparison of the vasodilatory effect between the intact (+E) and denuded (−E) epithelia groups showed that the vasodilatory effect induced by *DP* were significantly inhibited in the -E group (Fig. 1d). However, in the -E group, the inhibitory effect of Hy-*DP* treatment (36.69 ± 4.82%) was greater than the effect of *DP* treatment (17.55 ± 7.01%) (Fig. 1d and e). HDR-2-induced relaxation in rat aortic preparations was more significantly inhibited (55.77 ± 7.88%) than Hy-*DP*-induced relaxation by denudation of the endothelial layer. Interestingly, the relaxation induced by 5-*u*RCK was completely abolished in denuded aortic rings. These results indicated that HDR-2 exhibited a partial endothelium-dependent vasodilator effect.

### Effects of HDR-2 on NOS gene expression

**Fig. 2:**
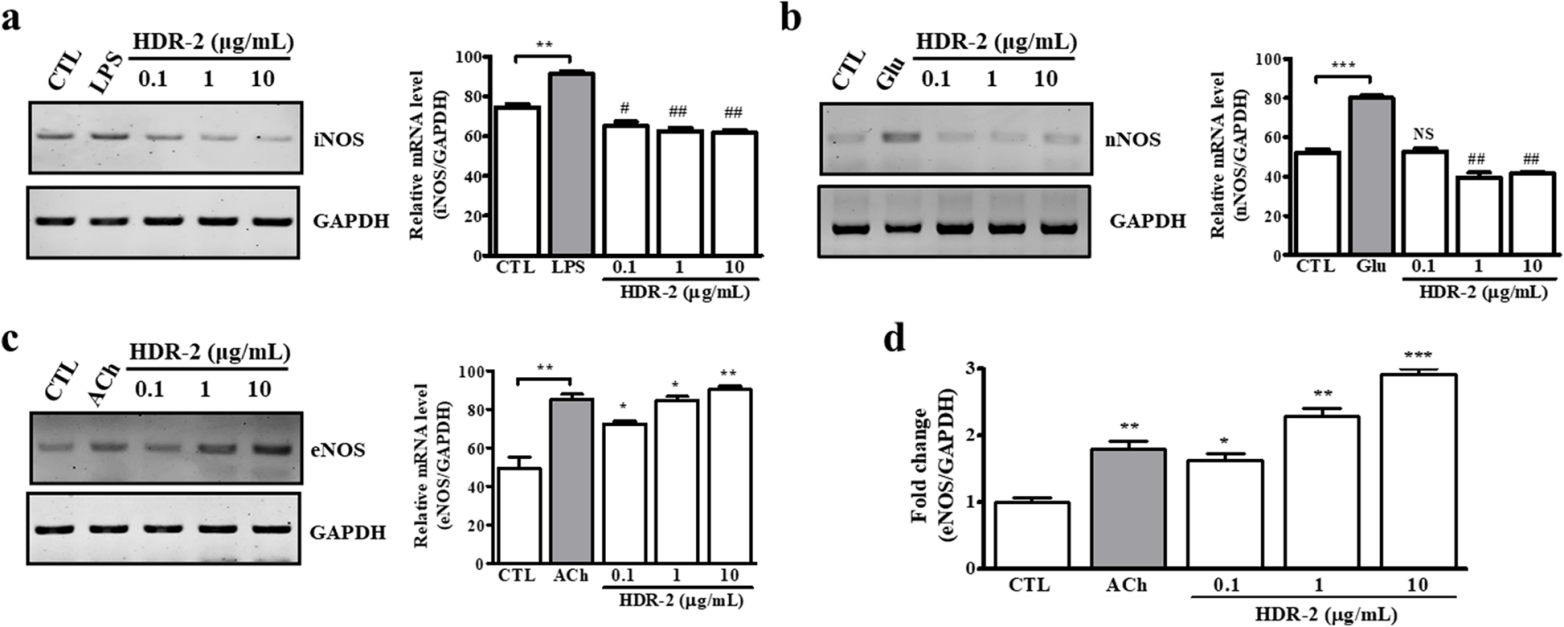
Effects of HDR-2 on the expression of NOS isoforms at the mRNA level (as assessed by RT-PCR). **a** RT-PCR analysis of iNOS mRNA expression in murine RAW 264.7 macrophages. Activated cultures were stimulated for 6 h with 1 μg/mL LPS or individual extracts before extraction of RNA. **b** RT-PCR analysis of nNOS mRNA expression in cultured rat hippocampal neurons. Activated cultures were stimulated for 6 h with 50 μM _L_-glutamate (Glu) or individual extracts before extraction of RNA. **c** RT-PCR analysis of eNOS mRNA expression in HUVECs. Activated cultures were stimulated for 6 h with 10 μM ACh or individual extracts before extraction of RNA. **d** eNOS mRNA expression was detected by real-time quantitative RT-PCR. ^*/#^*P* < 0.05, ^**/##^*P* < 0.01 and ^***/###^*P* < 0.001 vs the CTL group; NS, not significant. Symbols refer to significant differences (* symbol: increase/# symbol: decrease) between the treated group and the control group

As shown in Fig. 2a, iNOS mRNA was strongly expressed in lipopolysaccharide (LPS; from *Escherichia coli* 0111:B4)-stimulated RAW 264.7 cells. However, iNOS mRNA levels were not increased by treatment with HDR-2. In the expression of nNOS, as shown in Fig. 2b, nNOS mRNA was significantly increased by glutamate treatment in cultured hippocampal neurons, whereas HDR-2 treatment did not affect the expression of nNOS mRNA. We investigated the effects of HDR-2 on eNOS mRNA expression in HUVECs and verified that eNOS mRNA levels were significantly increased by HDR-2 treatment (Fig. 2c). We reconfirmed eNOS mRNA expression by real-time qPCR (Fig. 2d) and verified that the eNOS mRNA level of HUVECs was significantly increased in both ACh-treated cells and HDR-2 treated cells.

### HDR-2 stimulates the production of NO

**Fig. 3:**
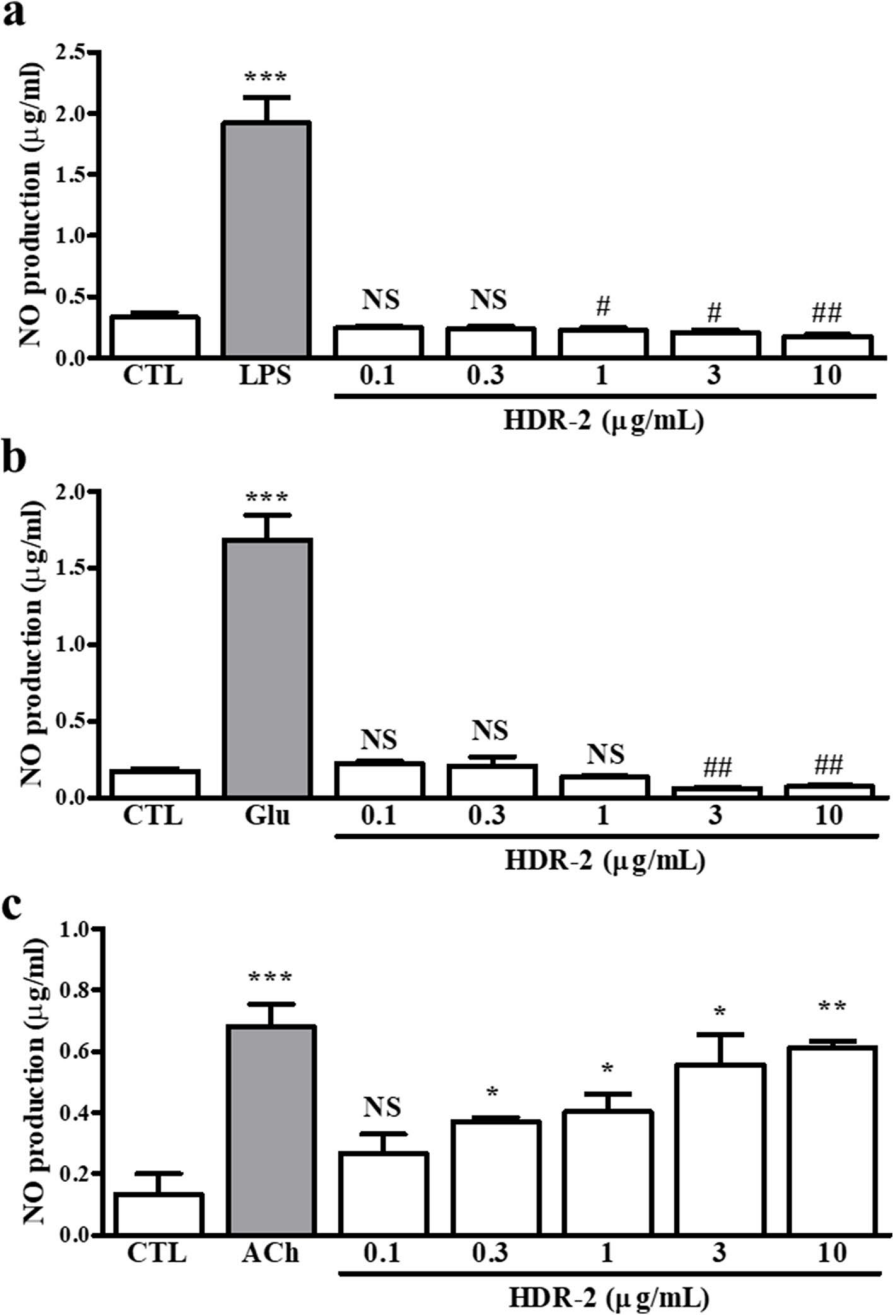
Effects of HDR-2 on NO levels. NO levels in murine RAW 264.7 macrophages, hippocampal neurons, and HUVECs treated with HDR-2. NO production at baseline (control; CTL) and after treatment with 1 μg/mL LPS or individual extracts in murine RAW 264.7 macrophages (**a**), at baseline and after treatment with 50 μM Glu or individual extracts in cultured rat hippocampal neurons (**b**), and at baseline and after treatment with 10 μM ACh or individual extracts in HUVECs (**c**). ^*/#^*P* < 0.05, ^**/##^*P* < 0.01 and ^***/###^*P* < 0.001 vs the CTL group; symbols refer to significant differences (* symbol: increase/# symbol: decrease) between the treated group and the control group. NS, not significant

To elucidate the relationship between the amount of NO production according to the eNOS expression effect of HDR-2, we verified the amount of NO produced in RAW 264.7 macrophages, cultured hippocampal neurons and HUVECs (Fig. 3). NO production was not changed by HDR-2 in cultured hippocampal neurons and RAW 264.7 macrophages, like the expression level of eNOS, but rather, NO production decreased as the concentration of HDR-2 increased. However, as shown in Fig. 3c, NO release was increased in a dose-dependent manner after HDR-2 treatment HUVECs, which may be related to the increased mRNA expression level of eNOS in HUVECs.

### HDR-2 decreased systolic blood pressure in 1 K-1C rats

**Fig. 4:**
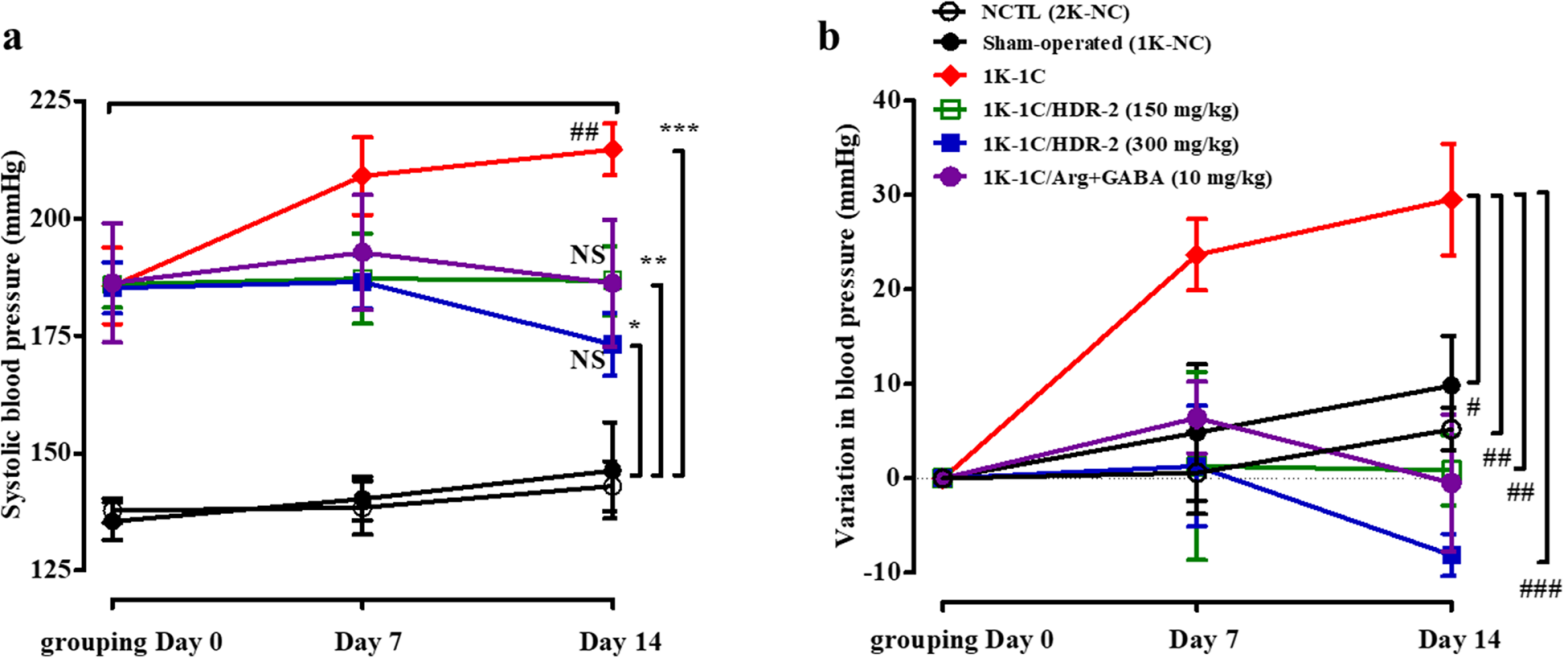
Effect of HDR-2 on systolic blood pressure (SBP) in 1 K-1C hypertensive rats at 0–2 weeks. Rats were intragastrically treated with saline, HDR-2, or a mixture of _L_-arginine and GABA, and SBP was measured using a NIBP system at 0, 1 and 2 weeks prior. Normal control (NCTL, 2 K-NC), sham-operated control (1 K-NC) and 1 K-1C control: rats were treated with saline. 1 K-1C/HDR-2: 1 K-1C rats were treated with HDR-2150 mg/kg BW or 300 mg/kg BW. 1 K-1C/Arg + GABA: 1 K-1C rats were treated with _L_-arginine (4 mg/kg BW) and GABA (6 mg/kg BW). Seven rats (*n* = 7) were included in each group. Values are expressed as the mean ± SD. *, ** and *** indicate *P* < 0.05, *P* < 0.01 and *P* < 0.001, respectively, compared with the sham-operated control (1 K-NC), #, ## and ### indicate *P* < 0.05, *P* < 0.01 and *P* < 0.001, respectively, compared with the 1 K-1C control group

**Fig. 5:**
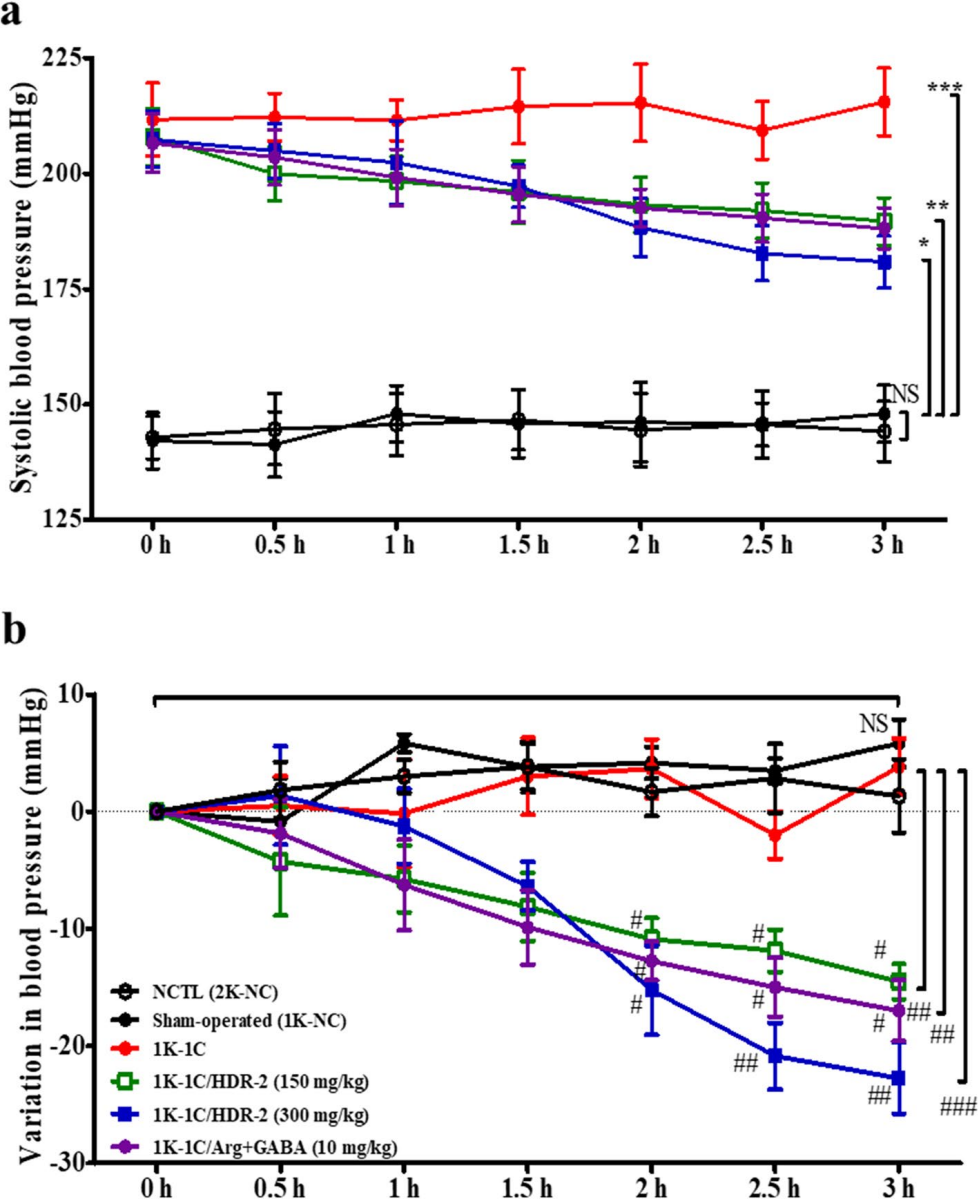
Acute effects of HDR-2 on changes in systolic blood pressure (SBP) in 1 K-1C hypertensive rat models. **a** Antihypertensive effects of different doses of HDR-2 in 1 K-1C rats within 3 h of administration with a single oral dose. **b** The decreasing amplitude of SBP of 1 K-1C rats at different time points after a single oral dose. Values are expressed as the mean ± SD. *, ** and *** indicate *P* < 0.05, *P* < 0.01 and *P* < 0.001, respectively, compared with the sham-operated control (1 K-NC), #, ## and ### indicate *P* < 0.05, *P* < 0.01 and *P* < 0.001, respectively, compared with the 1 K-1C control group

For the practical purpose of the utilization of HDR-2 as a physiological modulator, its antihypertensive activity was evaluated in vivo by measuring the variation in the SBP of 1 K-1C hypertensive rats over long-term intragastric administration (Fig. 4) and with an acute administration test (Fig. 5). Figure 4 shows the SBP change in all tested groups during the long-term intragastric administration experiment. The changes in the mean SBP of the 1 K-1C rats treated with HDR-2 for 2 weeks were measured using a tail-cuff method. The SBP values in the 1 K-1C group (214.67 ± 5.55 mmHg), 1 K-1C/HDR-2 groups (150 mg/kg; 186.71 ± 7.34 mmHg, 300 mg/kg; 173.14 ± 6.64 mmHg) and 1 K-1C/Arg + GABA group (186.16 ± 13.47 mmHg) were significantly higher than that of the sham-operated group (1 K-NC, 146.33 ± 10.27 mmHg) (Fig. 4a). However, the SBP values in the 1 K-1C/HDR-2 groups and 1 K-1C/Arg + GABA group at week two were significantly lower than those in the 1 K-1C group. The data were expressed as ΔSBP, which was the difference between the SBP on day 0 and the SBP measured one or 2 weeks after the beginning of the experiment. As shown in Fig. 4b, the ΔSBP values in the 1 K-1C-control group at weeks 1 (23.67 ± 3.78 mmHg) and 2 (29.50 ± 2.42 mmHg) were significantly higher (*P* < 0.01) than those in the sham-operated group (1 K-NC). The HDR-2-treated groups exhibited significant decreases in the mean ΔSBP observed in the 1 K-1C rats but did not demonstrate a change in heart rate compared with normotensive 1 K-NC rats and 1 K-1C rats (Table 1). The ΔSBP of the rats administered HDR-2 (300 mg/kg) was similar to that of rats on grouping day (0 day) until 1 week. After 2 weeks of treatment, the ΔSBP level of the HDR-2 (300 mg/kg) group was 8.14 ± 0.85 mmHg (*P* > 0.05, NS), which was lower than that of the 0-day rats (Fig. 4b). Moreover, 1 K-1C rats treated with the combination of _L_-arginine and GABA also exhibited a significant decrease in SBP compared to the 1 K-NC rats (*P* < 0.01) or 2 K-NC rats.

**Table 1:** Kidney and heart weights and heart rates of 1 K-1C hypertensive rats over two weeks. Data are expressed as the mean ± SD

Group	Number	Right kidney weight (mg)	Left kidney weight (mg)	Heart weight (mg)	Heart index (mg/g)	Heart rate (bpm)
**Control (2 K-NC)**	*n* = 7	1291.4 ± 9.4	1266.4 ± 26.4	1196.0 ± 42.4	3.8 ± 0.1	395.7 ± 30.8
**Sham-operated control (1 K-NC)**	*n* = 7	1331.6 ± 46.3	1285.2 ± 44.3	1380.6 ± 80.1	3.9 ± 0.1	379.5 ± 24.2
**1 K-1C**	*n* = 7	–	1626.3 ± 158.4	1299.6 ± 82.5	3.9 ± 0.2	380.8 ± 23.9
**1 K-1C/HDR-2 (150 mg/kg)**	*n* = 7	–	1422.2 ± 140.3	1149.2 ± 33.7	4.0 ± 0.3	381.4 ± 15.8
**1 K-1C/HDR-2 (300 mg/kg)**	*n* = 7	–	1455.7 ± 43.4	1282.6 ± 49.9	4.0 ± 0.1	377.3 ± 20.3
**1 K-1C/Arg + GABA (10 mg/kg)**	*n* = 7	–	1280.8 ± 132.2	1162.8 ± 135.1	4.1 ± 0.3	373.1 ± 26.3

### Antihypertensive action of single oral administration of HDR-2 in 1 K-1C rats

In the short-term test, the antihypertensive activity of HDR-2 was evaluated by measuring the SBP in 1 K-1C rats at 0, 0.5, 1, 1.5, 2, 2.5 and 3 h after oral administration, as shown in Fig. 5. Administration of saline to 1 K-1C rats caused no changes in SBP recorded from 0 h to 3 h following treatment. By contrast, half an hour after administration of HDR-2 (150 and 300 mg/kg BW) and a mixture of _L_-arginine and GABA (10 mg/kg BW), the blood pressures of the three groups had undergone a slight decrease. As shown in Fig. 5b, the maximum decline exhibited by the three groups was observed at 3 h, and the decreases in SBP were 14.50 ± 1.51 mmHg (HDR-2, 150 mg/kg), 22.75 ± 3.06 mmHg (HDR-2, 300 mg/kg) and 17.0 ± 2.62 mmHg (Arg + GABA, 10 mg/kg). SBP returned to the untreated initial levels within 24 h post administration (data not shown).

### Kidney and heart weight of 1 K-1C hypertensive rat models

Kidney and heart weights are shown in Table 1. The left kidney weights of the 1 K-1C group were significantly heavier than those of the control group (2 K-NC) and sham-operated group (1 K-NC). During the 2-week HDR-2 treatment, 150 mg/kg and 300 mg/kg treatment significantly reduced the increase in kidney weight in 1 K-1C rats. There were no differences in heart weight or heart index among all groups. Furthermore, all groups had similar heart rates.

### HDR-2 decreased ACE, AngII and NO levels in 1 K-1C rats in a long-term experiment

**Fig. 6:**
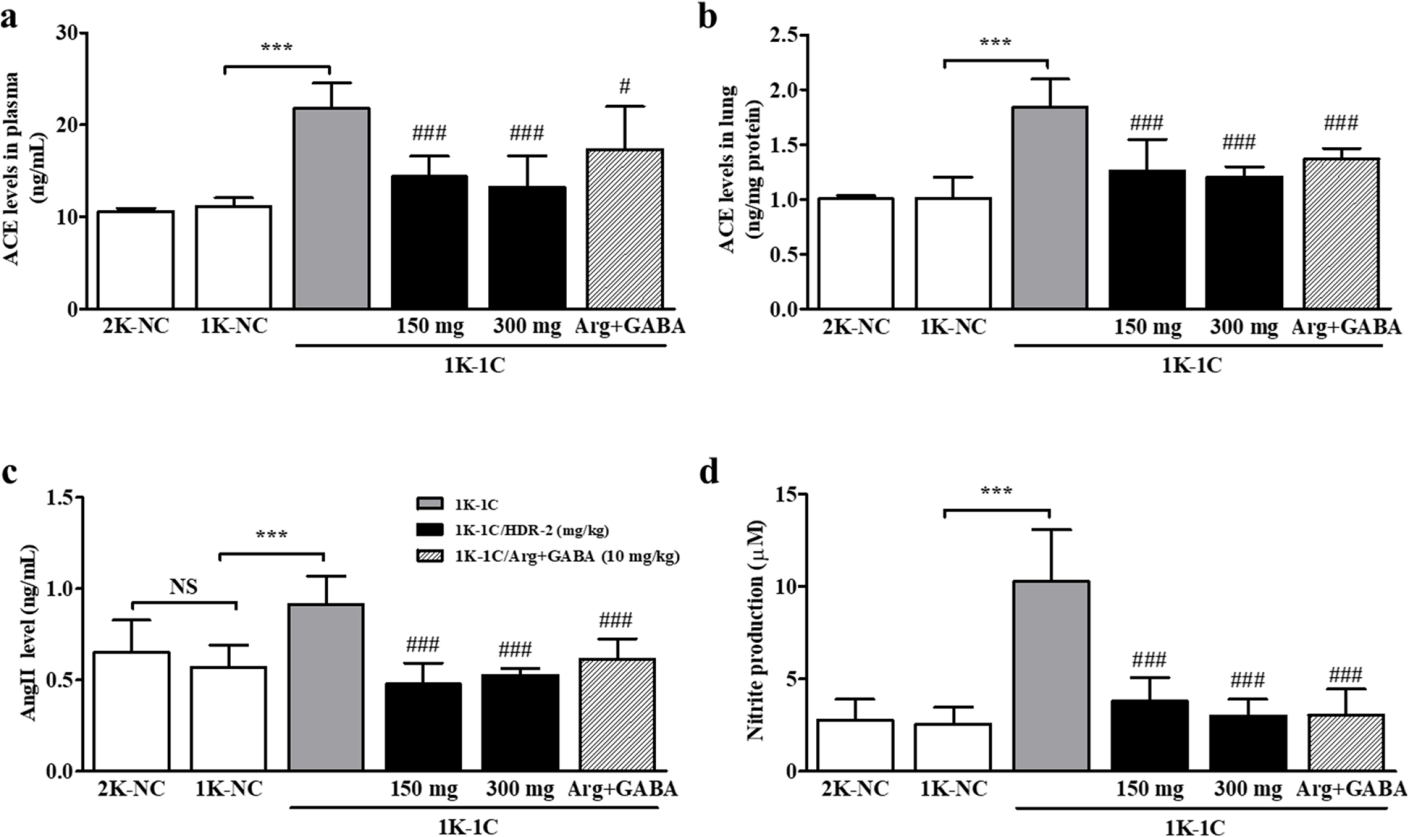
**a** Plasma ACE levels in the indicated treatment groups. **b** Lung ACE levels in the indicated treatment groups. **c** Plasma AngII levels in the indicated treatment groups. **d** Plasma nitric oxide (NO) levels in the indicated treatment groups. The values represent the means ± SD; ^***^P < 0.001, compared with the sham-operated control (1 K-NC) group; ^#^*P* < 0.05, ^##^*P* < 0.01 and ^###^*P* < 0.001, compared with the 1 K-1C control group

To further investigate the mechanism underlying the ACE inhibition activities of HDR-2 in 1 K-1C rats, the plasma and lung tissue ACE activities and the related plasma AngII levels were determined, and the results are presented in Fig. 6. The HDR-2 (150 mg/kg)- and HDR-2 (300 mg/kg)-treated groups exhibited significantly lower ACE activities in the plasma and lung tissue than the 1 K-1C rats (Fig. 6a, b). However, significant differences in the ACE activities between the 150 mg/kg and 300 mg/kg treated groups were not observed (*P* > 0.05). Moreover, the beneficial effect of HDR-2 in relieving hypertension was also evidenced by the reduction in AngII concentrations in plasma samples (Fig. 6c). Compared with control 1 K-1C rats, HDR-2-treated 1 K-1C rats demonstrated significantly reduced plasma AngII levels at 2 weeks after treatment (*P* < 0.001). The concentrations of AngII in 1 K-1C rat plasma samples were also significantly decreased in 1 K-1C rats treated with _L_-arginine and GABA (*P* < 0.001). As shown in Fig. 6d, the serum NO level was increased in the 1 K-1C hypertensive rat models. Treatment with HDR-2 (150 and 300 mg/kg) significantly decreased the generation of NO in serum compared to the 1 K-1C hypertensive control condition.

### HDR-2 decreased systolic blood pressure in SHR rats

**Fig. 7:**
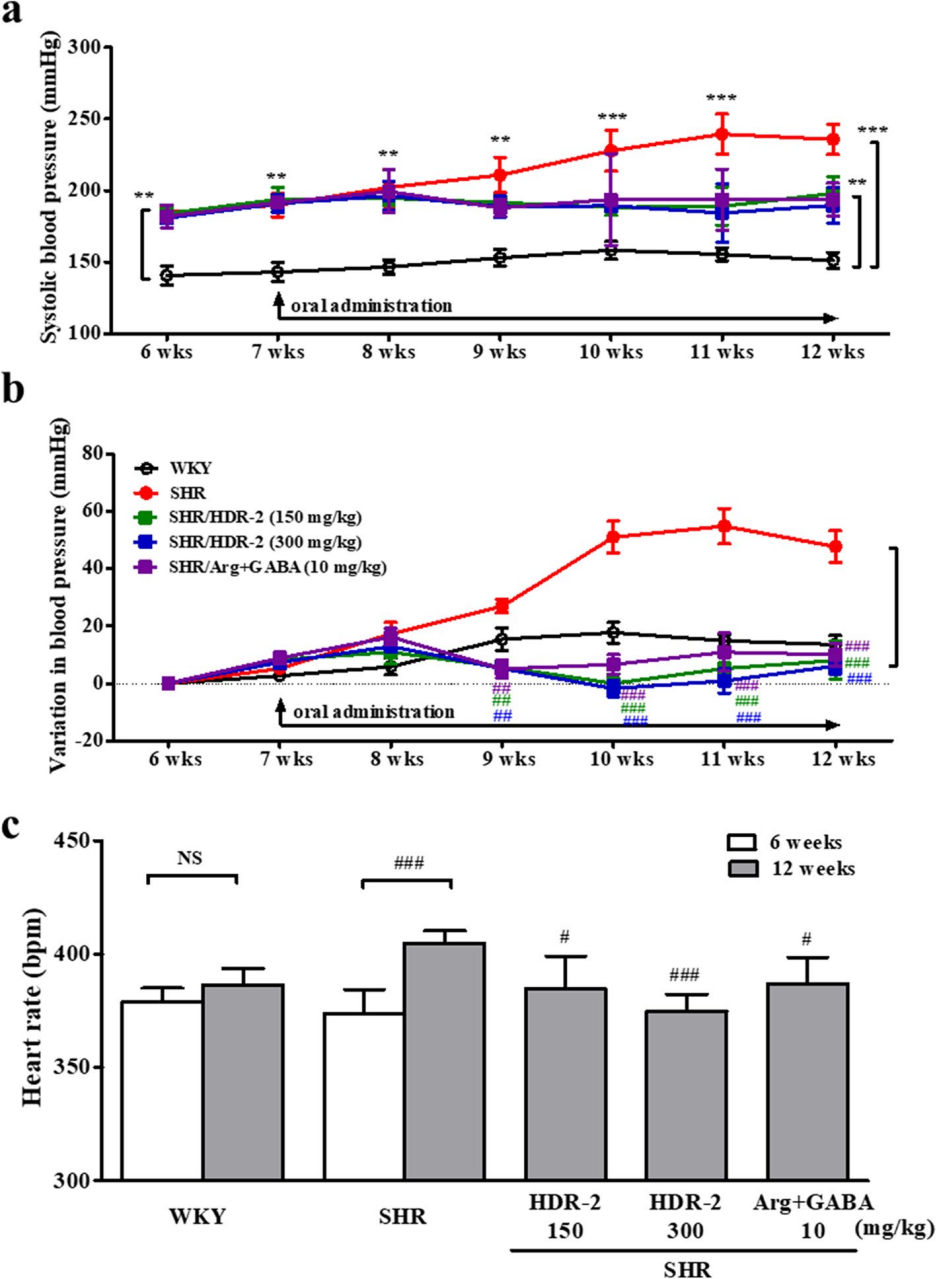
HDR-2 reduces blood pressure in spontaneously hypertensive rats (SHRs). Effects of long-term oral administration of HDR-2 on (**a**) systolic blood pressure (SBP, mmHg), (**b**) variation in blood pressure (ΔSBP, mmHg) and (**c**) heart rate (HR, bpm) as measured by tail-cuff plethysmography in SHRs. SHRs in the low-dose (150 mg/kg BW) and high-dose (300 mg/kg BW) groups were orally administered HDR-2 every day over a period of 5 weeks. SHRs in the combined _L_-arginine and GABA group (SHR/Arg + GABA) were given a mixture at 10 mg/kg BW once daily, whereas the SHRs and WKYs in the control groups were given saline (0.5 mL). Values are expressed as the mean ± SD. ** and *** indicate *P* < 0.01 and *P* < 0.001, respectively, compared with the WKY control group (WKY); ## and ### indicate *P* < 0.01 and *P* < 0.001, respectively, compared with the SHR control group. NS indicates not significant

The pathogenesis of hypertension in SHRs is similar to that in human essential hypertension [38]. Figure 7 shows the effect of the HDR-2 treatment, administered for 5 weeks, on SBP and heart rate in SHRs. Briefly, there were no significant differences among SHR groups at the beginning of the experiment, indicating that the baseline blood pressure of different SHR groups was the same. The SBP values in the SHR group (235.80 ± 10.59 mmHg), SHR/HDR-2 groups (150 mg/kg; 198.0 ± 11.79 mmHg, 300 mg/kg; 189.6 ± 12.58 mmHg) and SHR/Arg + GABA group (193.8 ± 11.39 mmHg) at week 12 were significantly higher than that of the normotensive WKY group (151.2 ± 3.63 mmHg) (Fig. 7a). As shown in Fig. 7b, however, oral administration of HDR-2 at two doses (150 mg/kg and 300 mg/kg) could significantly (*P* < 0.01) reduce the SBP in SHRs from the second week. Moreover, the SBP values in the two SHR/HDR-2 groups and the SHR/Arg + GABA group at week 12 were significantly lower (*P* < 0.001) than those in the SHR group. The data were expressed as ΔSBP, which was the difference between the SBP on day 0 and the SBP measured every week after the beginning of the experiment. The ΔSBP value in the SHR control group at week 12 (47.7 ± 2.22 mmHg) was significantly higher (*P* < 0.001) than that in the WKY group (13.5 ± 1.26 mmHg). The HDR-2-treated groups exhibited significant decreases in the mean ΔSBP in SHRs. Furthermore, the ΔSBP of the rats treated with HDR-2 (300 mg/kg) was similar to that of rats on grouping day (0 day) until 12 weeks (Fig. 7b). Interestingly, the _L_-arginine+GABA group also showed significant decreases in SBP compared with the SHR group (*P* < 0.001). The heart rate also significantly decreased in the HDR-2-treated groups after 5 weeks (aged 12 weeks) compared with the 12-week-old SHR control group (Fig. 7c). However, the heart rates of all HDR-2-treated groups were not significantly different from that of the WKY group (aged 6 weeks) or SHR control group (aged 6 weeks). As expected, the body weights of the rats in all groups increased over time, although no significant differences in body weight gain were observed among the groups during the six-weeks experimental period (Table 2). After sacrifice, the average weights of the heart, kidney, liver and spleen were not significantly different among the SHR groups. However, SHRs had a higher heart weight index, kidney weight index and liver weight index than WKYs, and these parameters were not altered by HDR-2 treatments. In fact, SHRs are characterized by relative increases in heart and kidney weights compared with normotensive WKY rats [39].

**Table 2:** Body, kidney, liver, spleen and heart weights of SHRs for six weeks. Data are expressed as the mean ± SD. ^**^P < 0.01 compared with the Wistar Kyoto rat (WKY) group

Group	Number	Body Weight (g)		Weight Index (mg/g of BW ratio)			
		Week-6	Week-12	Heart	Kidney	Liver	Spleen
**WKY**	*n* = 5	132.7 ± 9.35	319.3 ± 12.96	2.47 ± 0.06	5.84 ± 0.23	28.12 ± 1.61	2.68 ± 0.12
**SHR**	*n* = 5	123.2 ± 4.81	289.9 ± 6.58	4.19 ± 0.15^**^	7.57 ± 0.03^**^	40.68 ± 1.80^**^	1.78 ± 0.03^**^
**SHR/HDR-2 (150 mg/kg)**	*n* = 5	124.9 ± 5.27	289.7 ± 4.97	4.72 ± 0.03	7.53 ± 0.16	42.93 ± 2.72	1.76 ± 0.02
**SHR/HDR-2 (300 mg/kg)**	*n* = 5	121.2 ± 5.42	277.4 ± 4.37	4.24 ± 0.14	8.12 ± 0.20	43.83 ± 1.99	1.83 ± 0.03
**SHR/Arg + GABA (10 mg/kg)**	*n* = 5	122.8 ± 4.81	281.9 ± 2.38	4.26 ± 0.15	8.24 ± 0.15	42.77 ± 1.30	1.97 ± 0.02

### Antihypertensive action of single oral administration of HDR-2

**Fig. 8:**
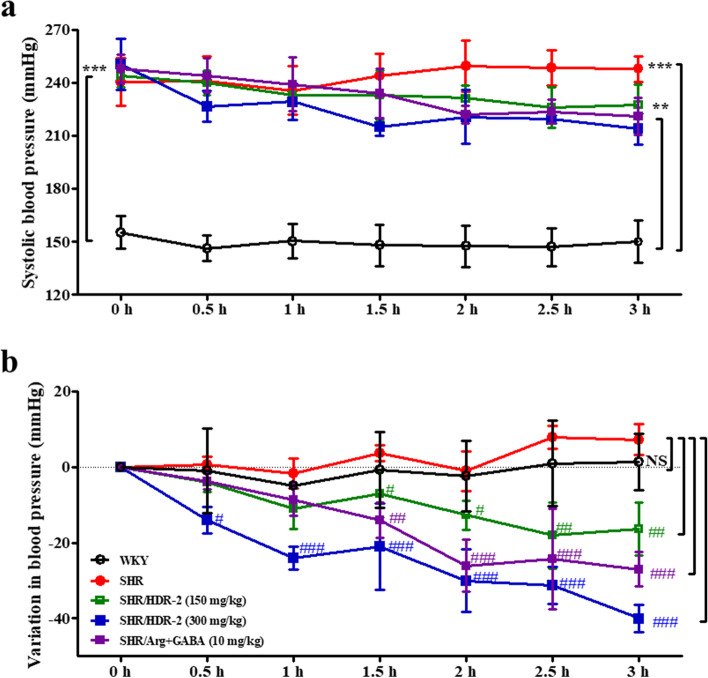
Changes in systolic blood pressure (SBP) of spontaneously hypertensive rats (SHRs) after a single oral administration of HDR-2. Single oral administrations were performed at a dosage of 150 mg HDR-2/kg body weight, 300 mg HDR-2/kg body weight and 10 mg mixture of _L_-arginine and GABA/kg body weight. SBP was measured 0, 0.5, 1, 1.5, 2, 2.5 and 3 h after administration. Values are expressed as the mean ± SD. *** indicates *P* < 0.001 compared with the WKY control group (WKY); #, ## and ### indicate *P* < 0.05, *P* < 0.01 and *P* < 0.001, respectively, compared with the SHR control group. NS indicates not significant

In the short-term test, the antihypertensive activity of HDR-2 was evaluated by measuring the SBP in SHRs at 0, 0.5, 1, 1.5, 2, 2.5 and 3 h after oral administration, as shown in Fig. 8. Half an hour after administration of HDR-2 (150 and 300 mg/kg BW) or a mixture of _L_-arginine and GABA (10 mg/kg BW), the blood pressure values of the three groups had undergone a slight decrease. Moreover, the lowering effect of HDR-2 on SBP showed an obvious dose-dependent relationship throughout the experimental period. The maximum decline exhibited by the three groups was observed at 2.5–3 h, and the decreases in SBP were 18.0 ± 8.71 mmHg (2.5 h), 40.0 ± 3.61 mmHg (3 h) and 27.0 ± 4.58 mmHg (3 h).

### Evaluation of the renin-angiotensin-aldosterone system (RAAS)

**Fig. 9:**
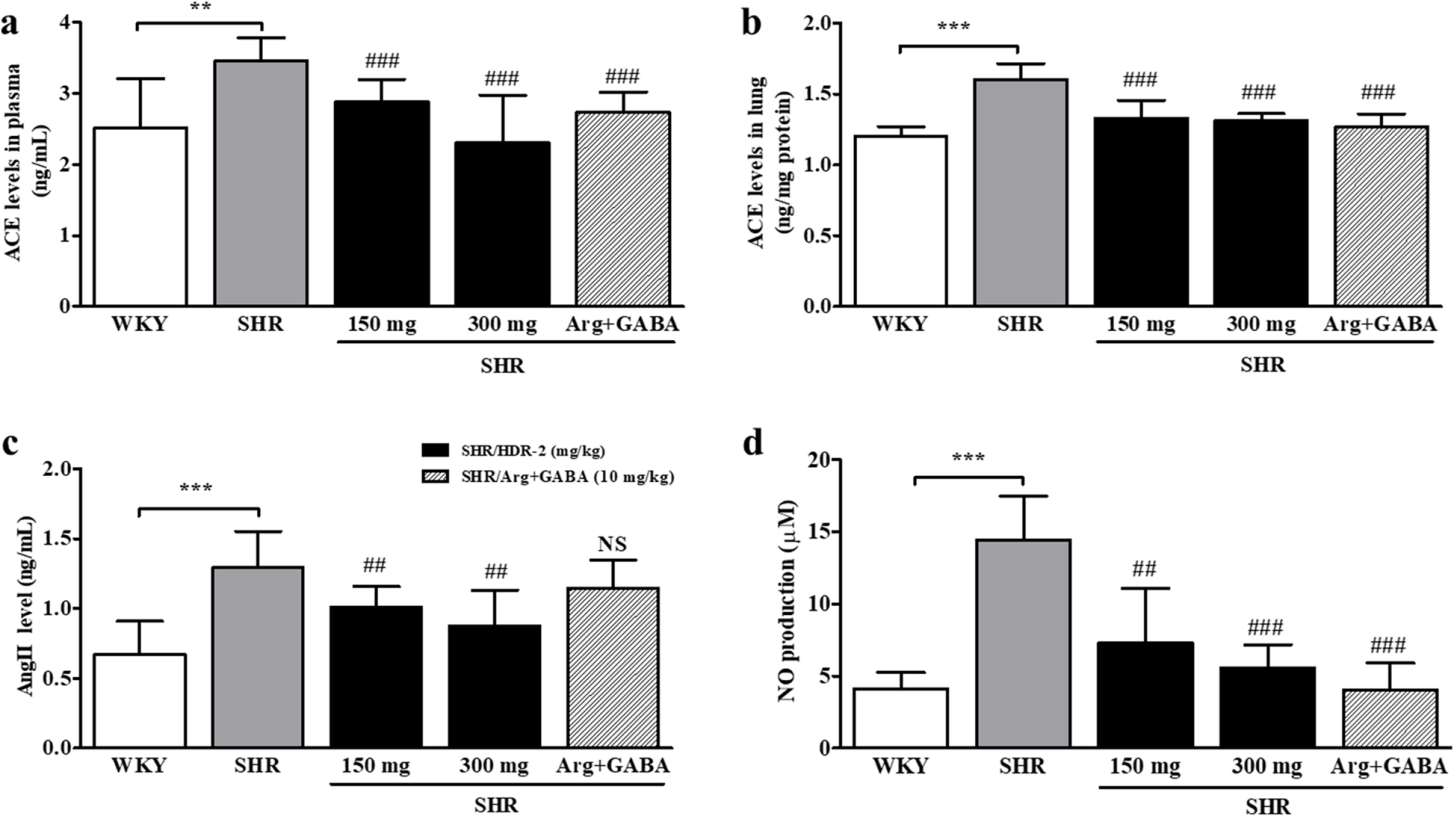
**a** Plasma ACE levels in the indicated treatment groups. **b** Lung ACE levels in the indicated treatment groups. **c** Plasma AngII levels in the indicated treatment groups. **d** Plasma nitric oxide (NO) levels in the indicated treatment groups. The values represent the means ± SD; ^**^P < 0.01 and ^***^P < 0.001, compared with the Wistar Kyoto rat (WKY) group; ^##^*P* < 0.01 and ^###^*P* < 0.001, compared with the spontaneously hypertensive rat (SHR)-control group

The in vivo ACE activities of plasma and lung tissue from the test groups were assessed 5 weeks after administration of HDR-2 (150 and 300 mg/kg BW) and a mixture of _L_-arginine and GABA (10 mg/kg BW) (Fig. 9a, b). Examination of plasma and lung ACE activity showed less activity in the HDR-2-treated groups and _L_-arginine+GABA-treated group than in the SHR control group. At the 150 mg/kg dose of HDR-2, the mean ACE levels in plasma and lung were 2.88 ± 0.32 ng/mL and 1.33 ± 0.12 ng/mg of protein, respectively. At the 300 mg/kg dose of HDR-2, the mean ACE levels in plasma and lung were 2.30 ± 0.67 ng/mL and 1.31 ± 0.05 ng/mg of protein, respectively. Moreover, at the 10 mg/kg dose of _L_-arginine+GABA, the mean ACE levels in plasma and lung were 2.73 ± 0.28 ng/mL and 1.27 ± 0.09 ng/mg of protein, respectively. In contrast, those of the SHR control group were 3.46 ± 0.32 ng/mL and 1.60 ± 0.11 ng/mg of protein, respectively. However, no significant differences were observed in the ACE activities between the 150 mg/kg and 300 mg/kg treated groups. All HDR-2 and _L_-arginine+GABA treatments significantly lowered ACE levels in plasma and lung tissue from SHRs (*P* < 0.001). Moreover, the beneficial effect of HDR-2 in relieving hypertension was also evidenced by the reduction in AngII concentrations in plasma samples (Fig. 9c). Compared with control SHRs, HDR-2-treated SHRs demonstrated significantly reduced plasma AngII levels at 5 weeks after treatment (*P* < 0.01). However, the concentrations of AngII in SHR plasma samples were not significantly decreased in SHRs treated with _L_-arginine and GABA (*P* > 0.05).

### The effect of HDR-2 on plasma NO production

We investigated plasma NO concentrations by HDR-2 administration (Fig. 9d). The plasma NO concentration in the SHR control rat was significantly increased compared with that in the WKY rat (14.42 ± 3.05 and 4.11 ± 1.13 μM, respectively; *P* < 0.001). Moreover, the NO concentrations in the SHR/HDR-2 (150 and 300 mg/kg) rats were significantly decreased (7.26 ± 3.82 and 5.57 ± 1.60 μM, respectively) in serum in a dose-dependent manner compared to those in the SHR control rat.

### HDR-2 stimulates the expression and phosphorylation of eNOS in aortic segments

**Fig. 10:**
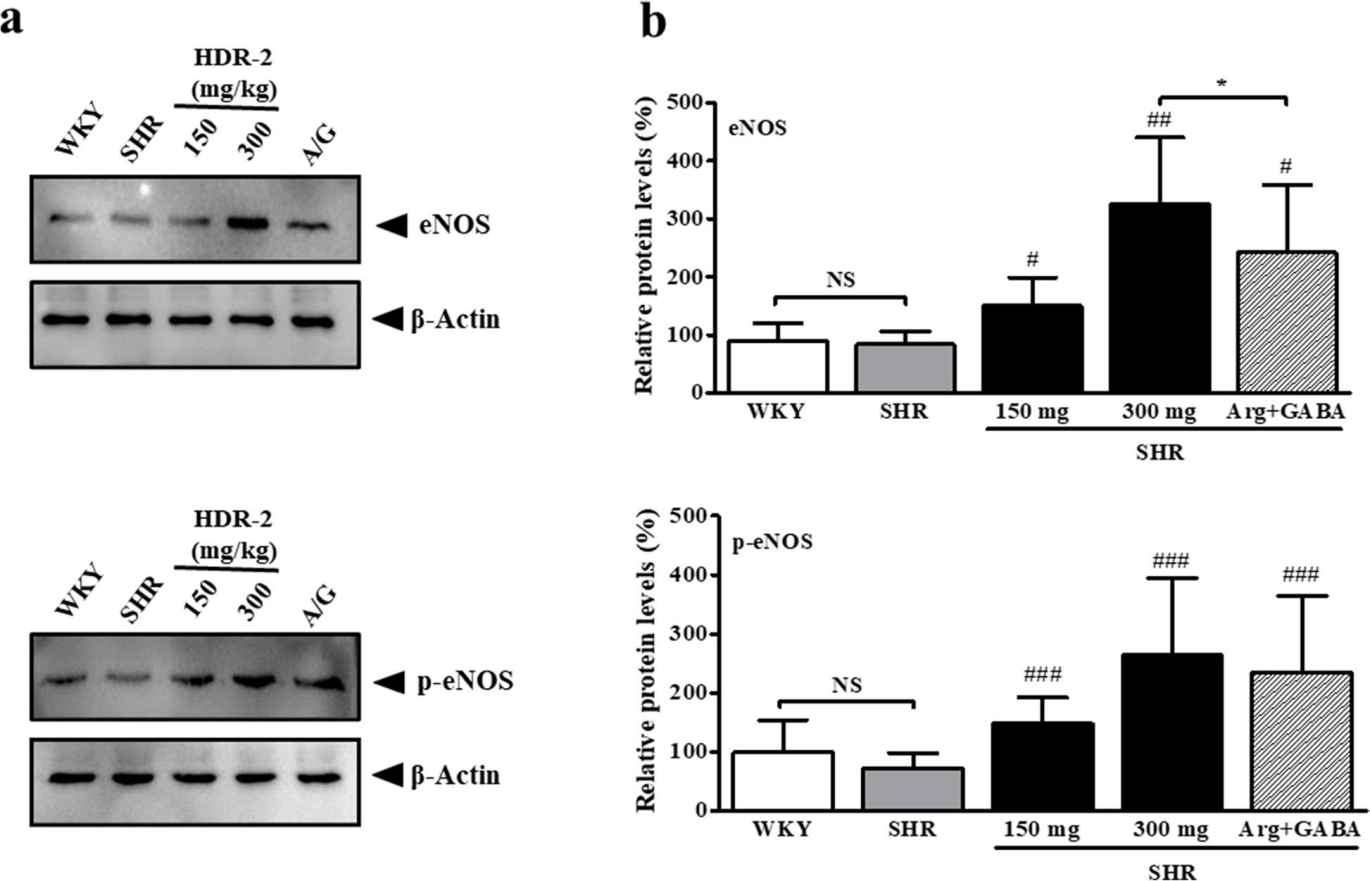
Effects of HDR-2 on the phosphorylation of eNOS (p-eNOS) and the expression of eNOS proteins in the aortae of the SHR control group and WKY group. **A** The protein expression and phosphorylation levels of eNOS in aortae were measured by western blotting. **B** The bar graphs indicate the average eNOS and p-eNOS levels. The data are expressed as the mean ± SD. *P* values for comparisons with SHR control groups are denoted as ^#^*P* < 0.05, ^##^*P* < 0.01 and ^###^*P* < 0.001, and those for comparisons with HDR-2 (300 mg/kg) groups are denoted as ^*^P < 0.05

eNOS serves an important basal regulatory function in the vasculature. When subjected to stimuli such as shear stress or ACh, eNOS, which is constitutively expressed in endothelial cells, oxidizes _L_-arginine to generate _L_-citrulline and NO. _L_-Arginine is one of the major biologically active components of Hy-*DP* and is regarded as the main compound responsible for its many pharmacological actions, including enhancement of eNOS expression, phosphorylation and NO production. We examined eNOS phosphorylation by western blotting analysis of tissue homogenate from the aortae of both WKY rats and SHRs after administration of HDR-2. As shown in Fig. 10, no significant difference in the level of eNOS expression was observed between samples from the aortae of the SHR control group and WKY group (*P* > 0.05). HDR-2 treatment increased the level of eNOS expression compared to that observed in the SHR control group (values of HDR-2 150 mg/kg and HDR-2 300 mg/kg were 150.6 ± 48.19% and 326.4 ± 113.90% higher than those of SHR control group, respectively). Administration of a mixture of _L_-arginine and GABA also stimulated the expression of eNOS in SHRs (*P* < 0.05). In addition, the efficiency of HDR-2 (300 mg/kg) to stimulate eNOS expression was much higher than that of the mixture of _L_-arginine and GABA in SHRs (*P* < 0.05).

In addition, our data show that Ser-1177 phosphorylation of eNOS (p-eNOS) is reduced in the aortae of the SHR control group (72.11 ± 25.99% lower than WKY group values; *P* > 0.05, NS). Treatment with HDR-2 increased the levels of eNOS phosphorylation relative to the levels observed in rats in the SHR control group (values of HDR-2 150 mg/kg and HDR-2 300 mg/kg were 148.9 ± 43.57% and 254.9 ± 129.30% higher, respectively, than those of SHR control group). The level of eNOS phosphorylation also increased in SHRs after administration of a mixture of _L_-arginine and GABA (*P* < 0.001).

### HDR-2 improved the vascular relaxation response in SHR aortae

**Fig. 11:**
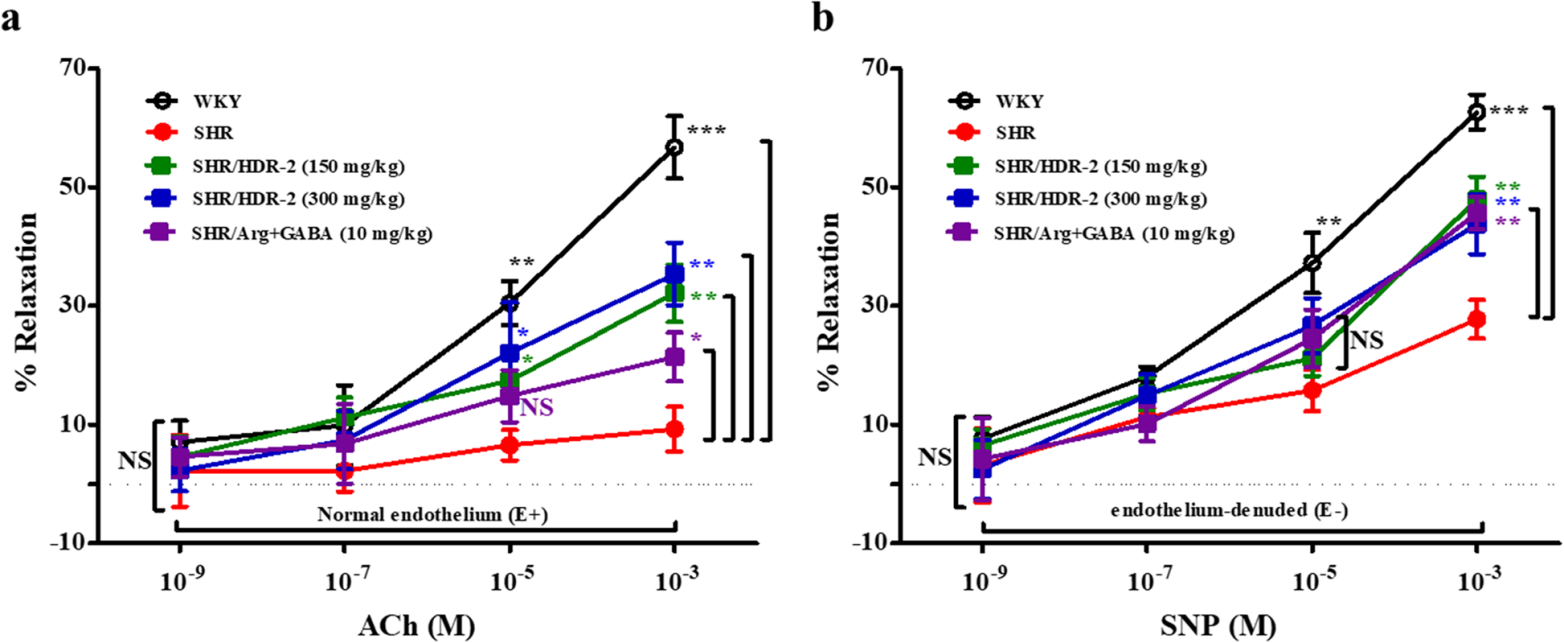
**a** Endothelium-dependent relaxation curve induced by acetylcholine (ACh) and (**b**) endothelium-independent relaxation curve induced by sodium nitroprusside (SNP) in thoracic aortic rings from the indicated treatment groups. The values represent the means ± SD; ^*^P < 0.05, ^**^P < 0.01 and ^***^P < 0.001, compared with the spontaneously hypertensive rat (SHR)-control group. NS indicates not significant

The effect of HDR-2 on vascular function in SHRs was evaluated as one of the possible mechanisms for its antihypertensive effects. To validate endothelium-dependent relaxation, we treated ACh (10^− 9^–10^− 3^ M) to aortic tissue that had been precontracted with PHE (10 μM). In the SHR control group, the relaxation response at 10^− 9^–10^− 3^ M ACh was significantly lower than that in the WKY control rats (*P* < 0.001) (Fig. 11a). In vivo treatment with HDR-2 at 150 and 300 mg/kg significantly improved the impaired ACh (1 mM)-induced relaxation in SHR aortae (*P* < 0.01, *P* < 0.01, respectively). The effective concentrations causing 50% relaxation (EC_50_) of SHR/HDR-2 (150 mg/kg) and SHR/HDR-2 (300 mg/kg) were 7.71 ± 0.21 μM and 7.18 ± 0.32 μM, respectively, which were significantly lower (*P* < 0.01) than that in the SHR control group (EC_50_ value: 15.66 ± 0.20 μM). Figure 11b shows the results of SNP-induced relaxation in each group of denuded aortic rings. SNP-induced endothelium-independent relaxation and the respective EC_50_ values were also significantly different among the SHR control group (26.67 ± 0.15 μM), SHR/HDR-2 (150 mg/kg) group (10.09 ± 0.16 μM) and SHR/HDR-2 (300 mg/kg) group (12.40 ± 0.14 μM). HDR-2 administration improved endothelium-dependent and endothelium-independent vasorelaxation in SHR thoracic aortae.

## Discussion

Our previous research has demonstrated that both Hy-*DP* and 5-*u*RCK have vasodilatory activities [24]. In the present study, we prepared a novel formulation, designated HDR-2, which contained 5-*u*RCK and Hy-*DP*, and administered it to two different animal models of hypertension. We found that HDR-2 treatment increased eNOS expression and phosphorylation and decreased blood pressure in hypertensive rats. These results indicate that HDR-2 administration has the potential to reduce blood pressure and prevent CVDs.

Both the vascular endothelium and vascular smooth muscle play important roles in regulating the function of the cardio-cerebrovascular system. Enhanced NO is a possible endothelium-dependent mechanism of vasodilation. As in our previous study, incubation of thoracic aortae with _L_-NAME, a NOS inhibitor, reduced vasodilation by both Hy-*DP* and 5-*u*RCK [24], suggesting a contribution of NO to relaxation in both groups. In this study, HDR-2-induced vasodilation was also reduced, but not completely inhibited, by treatment with _L_-NAME and ODQ in rat aortic rings with intact endothelium. Furthermore, removal of the endothelium from the aortic ring did not completely inhibit HDR-2-induced relaxation when the rings were preincubated with PHE. This finding suggests that HDR-2-induced relaxation involves both endothelium-dependent and endothelium-independent mechanisms, which is in accordance with our previous findings [24].

NO is a major endothelium-dependent relaxation factor, and its production by vascular endothelial cells plays a critical role in the regulation of vascular motor tone and the stability of blood flow as well as blood pressure [40, 41]. NO is synthesized by vascular endothelial cells using _L_-arginine as a substrate in a process catalyzed by NOS [42] and induces vascular smooth muscle relaxation by activation of guanylate cyclase [43]. Because it is an isoform of NOS that produces NO, eNOS also plays an important role in regulating systemic blood pressure [44]. The intracellular concentration of _L_-arginine normally greatly exceeds the level that is required for maximal enzyme kinetics of NOS; the Michaelis constant (Km) of _L_-arginine for NOS is 2.9 μmol/L, whereas the intracellular level of _L_-arginine in vivo is 0.8–2 mmol/L, with a plasma concentration in the range of 60–350 μmol/L [45, 46]. Nonetheless, _L_-arginine supplementation has been shown to improve endothelial function, reduce atherosclerosis progression and alter autonomic function, all consistent with enhanced NO synthesis [45, 47]. This is termed the _L_-arginine paradox and can probably be best explained by compartmentalization of NOS and a relative _L_-arginine deficiency rather than an absolute deficiency. The plasma _L_-arginine level was lower in the SHRs than in the WKY rats but was increased to a comparable level following _L_-arginine supplementation [48]. We developed and reported a technique for amplifying the _L_-arginine content of *DP* using the enzymatic hydrolysis method [16]. Furthermore, our previous study showed the effects of Hy-*DP* on vascular relaxation, eNOS expression and NO production in vitro [24]. In the present study, _L_-arginine contained in HDR-2 significantly decreased SBP in the hypertensive rat models and improved vascular function. However, the exact molecular mechanism of HDR-2-activated _L_-arginine transport remains to be further studied. Moreover, the results of the present study also showed that HDR-2 induces a dose-dependent chronic hypotensive effect on SHR, significantly reducing SBP as well as heart rate, which was significant at 150 and 300 mg/kg. For detailed mechanisms involving these results, HDR-2 should be examined for possible mechanisms using atropine (a nonselective muscarinic antagonist) and hexamethonium (an autonomic ganglion blocker). Therefore, it is hypothesized that the detailed mechanism underlying the heart rate reduction effect of HDR-2 treatment is based on the mechanisms described above.

The RAAS plays an important role in controlling blood pressure in the body [49]. In this system, ACE converts AngI to AngII, which increases blood pressure by causing the constriction of blood vessels [50, 51]. AngII also promotes oxidative stress, inflammation and fibrosis [52]. Furthermore, AngII is an important mediator of cardiovascular remodeling, which not only increases blood pressure but also enhances the generation of reactive oxygen species (ROS) [53]. Inhibition of ACE is a widely used strategy for the treatment of hypertension [54], so the food and pharmaceutical industries have an ongoing interest in new sources of ACE inhibitors with antihypertensive activity [55]. The SHR is considered to be a renin-independent model of hypertension in which plasma AngII levels are not elevated [56]. Nevertheless, ACE inhibitors have proven to be potent and effective agents in SHRs for the reduction in blood pressure and regression of several abnormalities, including cardiac and renal hypertrophy and endothelial dysfunction, that are developed in this model of genetic hypertension [57, 58]. This disparity can be understood if we consider that their antihypertensive effects are more closely related to the inhibition of tissue, rather than plasma, ACE [58, 59]. In fact, we found that chronic treatment with HDR-2 significantly reduced ACE activities in plasma and target organs for hypertension, such as lung tissue. Since HDR-2 was initially identified as an in vitro and in vivo ACE inhibitor, plasma AngII levels were assessed in both HDR-2-treated and untreated animals. Plasma AngII levels were significantly different between HDR-2-treated and untreated SHRs (Fig. 9c), indicating that HDR-2 may work as an ACE inhibitor in reducing blood pressure in hypertensive rats. Furthermore, in 1 K-1C hypertensive rats (regarded as a model of RAAS-dependent hypertension) (Fig. 6c), hypertension was also significantly responsive to HDR-2. In this study, plasma AngII levels in the 1 K-1C hypertensive rats and SHRs were significantly higher than those in the WKY normal control rats, suggesting that the RAAS was activated in the hypertensive model rats and confirming that the RAAS plays an important role in the pathological process of hypertension.

According to a previous studies, single oral administration of GABA at a concentration of 0.5 mg/kg to SHR significantly lowered systolic blood pressure [22], and the antihypertensive activity of GABA was significant at doses ranging from 0.05 to 5 mg/kg [23]. Our previous study reported that 100 mg of Hy-*DP* contains 2.8 mg of GABA, as estimated from the HPLC data [24]. We found that Hy-*DP* contains a sufficient concentration of GABA to decrease blood pressure in SHRs. Furthermore, the extracts *DP*, Hy-*DP* and 5-*u*RCK contained 2.61 ± 1.02 mg/g, 17.77 ± 1.36 mg/g and 1.63 ± 0.25 mg/g _L_-arginine, respectively. The HDR-2 samples used in this study contained _L_-arginine and GABA, and the ingested amounts in the in vivo administration test were 3.7 mg and 5.7 mg of _L_-arginine and GABA per kg∙BW, respectively. These studies showed that the blood pressure-lowering effect of these compounds is significant. Thus, _L_-arginine and GABA were potentially responsible for the antihypertensive effect. Although the mechanism underlying the hypotensive action of systemically administered GABA has not yet been fully elucidated, several hypotheses have been postulated. One of them postulates that because GABA rarely crosses the blood-brain barrier, this molecule acts not in the central nervous system but in the peripheral nervous system [60].

In this study, the chronic effective dose of HDR-2 in 1 K-1C rats and SHRs was 150–300 mg/kg BW per day. The chronic effective dose for an adult person weighing 60 kg was estimated to be 1.4–2.9 g/day of HDR-2, based on Kleiber’s law [61, 62]. However, it seemed to have a sufficient significant effect in the group treated with 150 mg/kg, so it can be expected to be effective even at lower concentrations. Therefore, further studies at lower concentrations are needed. HDR-2 can potentially serve as an important antihypertensive food or drug.

The expression and activity of NOS is altered in hypertensive animal models such as SHRs, and earlier studies have shown increased ROS, enhanced NOS expression, and NO production in SHRs [63]. In this study, the SHR control group also had higher basal plasma NO levels than the WKY group (Fig. 6d). Our results are consistent with previous studies showing that higher plasma NO levels in the SHR control group may reflect increased basal expression of iNOS [64] and oxidative stress-induced activation of iNOS [65]. The cause of vascular endothelial dysfunction of SHR is the increased activity of nicotinamide adenine dinucleotide phosphate oxidase (NADPH oxidase, NOX) in cells and the resulting oxidative stress induce iNOS and decrease NO bioavailability [66]. Furthermore, AngII stimulates ROS production mainly by activating NOXs in the vessel wall [67, 68]. Moreover, NOX seems to contribute to ROS production in SHR endothelial cells regardless of AngII [69]. Increased eNOS activity and decreased NOX activity were involved in the improvement of vascular endothelial function in SHR [70], and the increase in eNOS expression and activation was strongly associated with elevated plasma NO levels in SHR [71]. Notably, a reduction in eNOS activity is known to be involved in the hypertensive state of SHR [72]. Consistent with the published information, our data show that eNOS phosphorylation is slightly reduced in the aortae of SHRs. Moreover, we found that HDR-2 significantly increased eNOS phosphorylation in the aortae of SHRs. Reduced eNOS and AKT phosphorylation are simultaneously present in the aortae of SHRs [73]. In this study, however, plasma total NO levels and eNOS expression and phosphorylation levels were assessed merely as a control for hypertension-associated parameters. Therefore, further studies are required to confirm our findings.

Incubation of thoracic aortae with _L_-NAME, an NOS inhibitor, reduced vasodilation in HDR-2, suggesting a contribution of NO to relaxation in HDR-2-treated groups (Fig. 1c). This NO-dependent vasodilation may be due to the enhanced NO bioavailability through scavenging of free radicals or increased NO production in the vasculature. Vascular relaxation due to SNP, an exogenous NO donor, was also significantly enhanced in the HDR-2-treated group compared to the untreated SHR group (Fig. 11b). This implies the involvement of either an endothelium-independent mechanism (in addition to endothelium-dependent mechanisms) or enhanced NO bioavailability in the HDR-2-treated group compared to the untreated SHR group.

In this study, HDR-2 treatment improved ACh-induced endothelium dependence in isolated intact SHR thoracic aortae and SNP-induced endothelium-independent relaxation in isolated denuded SHR thoracic aortae. However, although these findings suggest that HDR-2 is involved in blood pressure regulation in a partially endothelium-independent manner, it is currently unclear whether HDR-2 is able to directly regulate vascular smooth muscle cell (VSMC) phenotypic switching and proliferation in hypertension. A recent study reported that transient receptor potential vanilloid 1 (TRPV1) cation channel activation elevates the phosphorylation of PKA and eNOS in endothelial cells and plasma NO concentration, improves endothelium-dependent relaxation in mesenteric arteries, and lowers arterial pressure in genetically hypertensive rats [74, 75]. Thus, endothelial TRPV1 activation can be considered a potential strategy for the management of hypertension. To fill these gaps, therefore, we will investigate the detailed molecular mechanism of HDR-2 on phenotypic switching and proliferation of VSMCs and chronic activation of TRPV1 in hypertension.

## Conclusions

The present study, to the best of our knowledge, is the first to compare the effects of long-term treatment with HDR-2 on vascular function in a 1 K-1C hypertensive rat model and SHRs through several mechanisms. The main finding of the study was that reduced blood pressure was concomitant with increased vasodilation, reduced nitrosative stress, ACE and AngII, and enhanced eNOS expression. While our findings are novel, further research is needed to ascertain the role of HDR-2 in mechanisms involved in endothelium-independent vasorelaxation to achieve a comprehensive understanding of the underlying mechanisms. The findings from this study may establish the potential of natural products in the management of hypertension and associated complications.

## Data Availability

All data and analyses in the current study are available from the corresponding author upon reasonable request.

## Abbreviations

ACE: Angiotensin-converting enzyme
ACh: Acetylcholine chloride
cAMP: Cyclic 3′,5′-adenosine monophosphate
cGMP: Cyclic 3′,5′-guanosine monophosphate
CVD: Cardiovascular disease
*DP*: *Dendropanax morbiferus* H. Léveille extract
eNOS: Endothelial nitric oxide synthase
EC_50_: Half maximal effective concentration
GABA: γ-Aminobutyric acid
GAPDH: Glyceraldehyde 3-phosphate dehydrogenase
GC: Guanylate cyclase
HDR-2: Composed of Hy-*DP* and 5-*u*RCK in a 2:1 mass ratio
HUVEC: Human umbilical vein endothelial cell
Hy-*DP*: Enzymatically hydrolyzed *Dendropanax morbiferus* H. léveille extract
IC_50_: Half maximal inhibitory concentration
iNOS: Inducible nitric oxide synthase
_L_-NAME: N^ω^-nitro-_L_-arginine methyl ester
NO: Nitric oxide
NOS: Nitric oxide synthase
nNOS: Neuronal nitric oxide synthase
ODQ: 1H-[1,2,4]oxadiazolo[4,3-a]quinoxalin-1-one
PDE: Phosphodiesterase
PHE: Phenylephrine hydrochloride
SHRs: Spontaneously hypertensive rats
1 K-1C: 1-Kidney, 1-clip goldblatt hypertensive rats
5-*u*RCK: 5% Unripe *Rubus coreanus* miquel ethanol extract
